# The Effectiveness of Manuka Honey in Treating *Staphylococcus aureus* in *C. elegans*

**DOI:** 10.17912/micropub.biology.002081

**Published:** 2026-04-21

**Authors:** Riley Lesko, Olivia Long, Kelsey Murphy

**Affiliations:** 1 University of Pittsburgh at Greensburg, Greensburg, Pennsylvania, United States

## Abstract

Antibiotic resistance is a severe problem stemming from the overuse of antibiotics. Manuka honey, a unique honey from New Zealand, may serve as an alternative to traditional antibiotics for treating bacterial infections. A transgenic line of *Caenorhabditis elegans* , *hlh-30* ::3xFLAG::eGFP, was utilized as a stress reporter strain to quantify changes in GFP expression associated with host response to *S. aureus* infection and honey treatment. Imaging, longevity, and developmental assays all demonstrated that manuka honey reduced the host stress response to *S. aureus* infection in *C. elegans* . These findings suggest that manuka honey may be effective against *S. aureus* infections.

**Figure 1. Manuka honey’s effects on S. aureus infection in C. elegans stress response, longevity, and development:** A) Green fluorescent protein (GFP) fluorescence imaging of *hlh-30* ::3xFLAG::eGFP (referred to as strain VZ892) adult animals. Exposure to *S. aureus* resulted in increased fluorescence, with bright accumulations along the intestinal tract of the worms. Treatment with manuka honey helped return fluorescence to the original diffuse baseline. B) Graph of the average corrected total fluorescence (ACTF) from imaging. After conducting a one-tailed t-test, there was a significant difference between the control group and worms exposed to *S. aureus* ( *P* = 0.0006). There was also a significant difference between worms exposed to *S. aureus* and worms exposed to *S. aureus* followed by treatment ( *P* = 0.0055). There was no significant difference between the control and treatment groups ( *P* = 0.1109). C) Kaplan-Meier survival curve for VZ892 animals. The control group exhibited 50% lethality at 192 hrs. Animals exposed to *S. aureus* exhibited 50% lethality at 24 hrs. VZ892 animals exposed to *S. aureus* and manuka honey treatment exhibited 50% lethality at 72 hrs. A log-rank (Mantel-Cox) test showed a significant difference among all conditions ( *P* = 0.0025). D) Kaplan-Meier survival curve for N2 animals. The control group exhibited 50% lethality at 180 hrs. Animals exposed to *S. aureus* exhibited 50% lethality at 48 hrs. N2 exposed to *S. aureus* and manuka honey treatment had 50% lethality at 84 hrs. A log-rank (Mantel-Cox) test showed a significant difference among all conditions ( *P* < 0.0001). E) A statistically significant difference in development was observed in worms exposed to *S. aureus* compared to their respective control groups (N2 *P* = 0.0002, VZ892 *P* = 0.0005), as well as between *S. aureus* exposed groups and manuka honey treatment groups (N2 *P* = 0.0009, VZ892 *P* = 0.0015). Significance was determined using a one-tailed t-test.

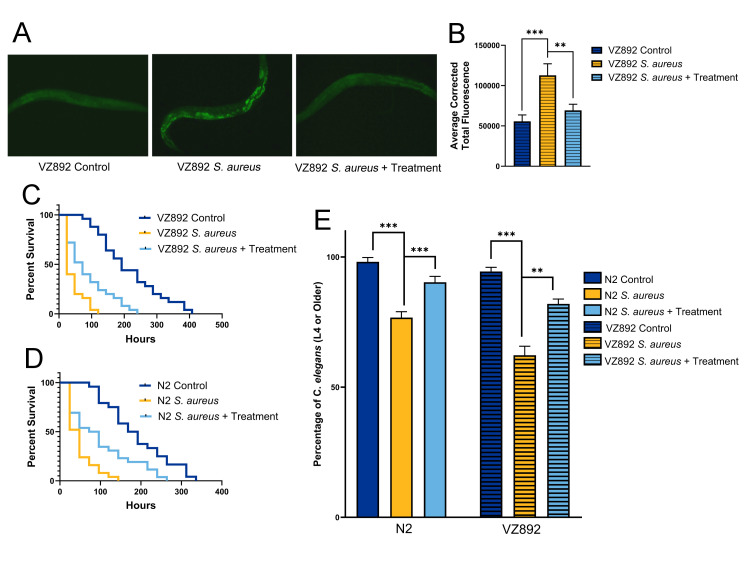



## Description

Antibiotic resistance is a major public health concern, with *Staphylococcus aureus* strains evolving rapidly and becoming less responsive to standard treatments (Aslam et al., 2018; Myles and Datta, 2012). In the United States, there are approximately 2.8 million antibiotic-resistant cases per year, resulting in over 35,000 deaths (Myles and Datta, 2012). In an effort to combat antibiotic resistance, alternative antibacterial treatments that do not promote resistance are being explored. Manuka honey is one such potential treatment. It is produced exclusively in New Zealand from the nectar of the manuka tree, *Leptospermum scoparium* (Sultanbawa, 2014). This honey is well known for its strong antibacterial and antifungal properties, rivaling those of other honeys (Almasaudi et al., 2017). The quality of the manuka honey is measured by methylglyoxal (MGO) concentrations (Sultanbawa, 2014), which is rated using the Unique Manuka Factor (UMF) scale. Lower UMF values correspond to lower MGO concentrations and, therefore, reduced antibacterial activity (Atrott and Henle, 2009). The complex interactions between MGO and peroxides in honey prevent pathogens from developing resistance to manuka honey (Sultanbawa, 2014). Manuka honey has been shown to be effective in treating *S. aureus* infections by reducing the bacterial growth (Almasaudi et al., 2017). However, its effects have not been explored in a *C. elegans* model.

This study utilized *Caenorhabditis elegans* , a transparent nematode widely used as a model organism. *C. elegans* is a microscopic worm with a short lifespan of approximately two to three weeks (Apfeld and Alper, 2018). From the egg stage, it progresses through several larval stages before reaching the phenotypically distinct L4 stage, after which it matures into an adult (Corsi, 2006). These worms are especially robust in the lab and can be easily and cost-effectively maintained on media plates seeded with a lawn of *Escherichia coli* . Previous studies have used *C. elegans* as a model for both methicillin-susceptible and methicillin-resistant *S. aureus* (MSSA and MRSA, respectively) infections (Thompson and Brown, 2014). The bacteria consumed by the worm colonize the intestinal tract (Sifri et al., 2003), leading to degradation of the digestive system and eventual death of the organism (Irazoqui et al., 2010).

This research investigates the use of *C. elegans hlh-30* ::3xFLAG::eGFP (strain VZ892 ) as a model for *S. aureus* infection and its responsiveness to manuka honey treatment (Martina et al., 2021). Both the wild-type *C. elegans* strain N2 and the stress reporter strain VZ892 were used to evaluate the effects of manuka honey on infection outcomes (Martina et al., 2021). VZ892 contains a GFP-tagged version of the endogenous *hlh-30* locus and is phenotypically similar to wild-type. This strain serves as a reporter of cellular stress and immune activation. Upon exposure to stressors such as *S. aureus* , *hlh-30* translocates, and GFP fluorescence increases, enabling visualization and quantification of the host response (Colino-Lage et al., 2024). Worms were divided into three groups: control (no *S. aureus* , no manuka honey treatment), *S. aureus* only, and *S. aureus* with manuka honey treatment (UMF 20). Assays included GFP fluorescence imaging, lifespan analysis, and developmental progression.

To utilize the stress reporter strain VZ892 , an imaging assay was performed to quantify GFP expression. Localized GFP fluorescence within the intestinal tract suggests activation of host defense responses at the primary site of *S. aureus* colonization. VZ892 worms in the control groups had a mean fluorescence of 55,768.3 ACTF, with fluorescence diffusely distributed throughout the worms' bodies ( Figure 1A, left panel). Exposure to *S. aureus* resulted in a 102.2% increase in fluorescence to 112,770 ACTF. In addition to this increase, fluorescence became localized, with intense pinpoint accumulations in the digestive system ( Figure 1A, middle panel). Following treatment with manuka honey ( Figure 1A, right panel), fluorescence decreased by 38.5% to 69,296 ACTF as compared to infected worms. No significant difference was found between the treatment group and the control group following a one-tailed t-test ( *P* = 0.1109). Furthermore, upon exposure to manuka honey, the GFP fluorescence returned to a diffuse pattern similar to that observed in control worms.

To assess survival, a longevity assay was performed. Lifespans of both the VZ892 ( Figure 1C ) and N2 ( Figure 1D ) strains were significantly reduced following exposure to *S. aureus,* consistent with previous findings (Sifri et al., 2003). The VZ892 LT50 decreased 85.7% from 168 hours to 24 hours. Similarly, the N2 LT50 decreased by 73.3% from 180 hours to 48 hours.

Treatment with manuka honey improved lifespans but did not restore them to baseline levels. With treatment, the VZ892 strain LT50 improved to 72 hours (a 200% increase), and the N2 strain LT50 improved to 84 hours (a 75% increase).

Previous studies suggest that exposure to *S. aureus* &nbsp;represses development in *C. elegans* , resulting in delayed growth (Sifri et al., 2003). To determine whether manuka honey could mitigate this effect, a developmental assay was conducted to assess the percentage of worms reaching the L4 larval stage or adulthood within a normal timeframe. Figure 1E shows that both VZ892 and N2 control groups had high rates of normal development (94.42% and 98.15%, respectively). Exposure to *S. aureus* significantly reduced these rates to 62.26% for VZ892 and 76.74% for N2 . Treatment with manuka honey improved development to 82.01% for VZ892 &nbsp;and 90.30% for N2. These results indicate that *S. aureus* exposure slows the development in *C. elegans* and that manuka honey treatment partially restores normal developmental progression.

These findings support the use of *C. elegans* as a model for screening natural therapeutics such as manuka honey. Additionally, the results suggest that UMF 20 manuka honey can reduce *S. aureus* -induced stress and pathogenesis in this model, indicating its potential as an alternative or adjunct to traditional antibiotics. Further studies are warranted to explore dose dependence across different UMF values, long-term effects, and efficacy against resistant *S. aureus* strains such as MRSA.

## Methods

***C. elegans* care**

N2 and  VZ892  *C. elegans* strains were obtained from the Caenorhabditis Genetics Center (CGC). Strains were maintained at 21 °C on 60 mm plates of NGM Lite (USBiological Life Sciences) seeded with 50 μL of *E. coli * OP50 (NGM/ OP50 ). Worms were transferred to fresh NGM/ OP50 plates using a sterilized platinum pick as needed to prevent starvation. All experiments utilized 2-day-old adult worms to avoid the confounding effects of *S. aureus* on worms' development.

***S. aureus* solution**

A single *S. aureus* (ATCC 14775) colony was isolated and grown in a tryptic soy broth (TSB). Cultures were incubated shaking at 37 °C for 24 hours. Fresh cultures were prepared from a single colony as needed for seeding experimental plates.

***S. aureus* plates**

Worms were infected with *S. aureus* by exposure to bacterial lawns grown on tryptic soy agar plates. Lawns were prepared by seeding 35 mm plates with 5 μL of the diluted culture (50% overnight *S. aureus* culture and 50% fresh TSB). Plates were incubated at 37 °C for 24 hours or until a lawn was visible.

**Manuka honey solution**

A 135% (w/v) solution of 20 UMF manuka honey and distilled water was vortexed until fully dissolved, with no visible residue stuck to the sides of the container. The solution was made fresh as needed. For treatment plates, 50 μL of the solution was spread onto an NGM Lite plate seeded with OP50 using a sterile spreader. Plates were allowed to dry for 24 hours in a biological safety cabinet.

**Infecting *C. elegans* with *S. aureus* and treating with manuka honey**

Two-day-old adult worms were placed on OP50 control plates or TSA plates containing *S. aureus* lawns for 24 hours. Following exposure, half of the worms from *S. aureus* plates were transferred to manuka honey treatment plates, while the remaining worms were transferred to OP50 plates. Exposure to treatment or control conditions was maintained for 24 hours, after which worms were imaged, assessed for longevity, and subjected to developmental assays.

**Imaging Assay**

Slides were prepared using a 3% agarose pad. A 25 μL drop of 100 mM sodium azide was added to the agarose pad to which worms were placed. Imaging was performed using a Zeiss Primo Star microscope with a Moticcam Pro camera. Images were captured using the Motic Image Plus 3.0 software at 100x total magnification with a 480 nm fluorescence filter. Images of the entire worm were then analyzed with ImageJ version 1.54g following the previously described protocol (Fitzpatrick, 2014). Three independent trials were conducted with fifteen worms per experimental group.

**Longevity Assay**

After 24-hour exposure to *S. aureus* , twenty worms were transferred to OP50 plates and counted daily. Death was determined through lack of response upon prodding the worms with a sterilized pick. Dead worms were removed and recorded daily. The assay continued until all worms were deceased and was performed in triplicate.

**Developmental Assay**

Twenty adult animals were transferred to plates containing *S. aureus* with or without treatment and allowed to lay eggs for 5 hours. Adults were removed, and eggs were left to develop for 48 hours at 21 °C. After 48 hours, offspring were staged and counted to assess developmental progression. Developmental assays were completed in triplicate.

## Reagents

Strains:

**Table:** 

**STRAIN**	**GENOTYPE**	**AVAILABLE FROM**
N2	C. *elegans* wild isolate	Caenorhabditis Genetics Center (CGC)
VZ892	*hlh-30* ::3xFLAG::eGFP	CGC
